# Novel insight into the therapeutical potential of flavonoids from traditional Chinese medicine against cerebral ischemia/reperfusion injury

**DOI:** 10.3389/fphar.2024.1352760

**Published:** 2024-02-29

**Authors:** Jing Zhou, Feiyue Sun, Wenli Zhang, Zhitao Feng, Yi Yang, Zhigang Mei

**Affiliations:** ^1^ Key Laboratory of Hunan Province for Integrated Traditional Chinese and Western Medicine on Prevention and Treatment of Cardio-Cerebral Diseases, College of Integrated Traditional Chinese Medicine and Western Medicine, Hunan University of Chinese Medicine, Changsha, Hunan, China; ^2^ School of Pharmacy, Hunan University of Chinese Medicine, Changsha, Hunan, China; ^3^ Third-Grade Pharmacological Laboratory on Chinese Medicine Approved by State Administration of Traditional Chinese Medicine, College of Medicine and Health Sciences, China Three Gorges University, Yichang, Hubei, China; ^4^ The First Affiliated Hospital of Hunan Traditional Chinese Medical College, Zhuzhou, Hunan, China

**Keywords:** flavonoid, traditional Chinese medicine (TCM), cerebral ischemia/reperfusion injury (CIRI), ischemic stroke, neuroprotection

## Abstract

Cerebral ischemia/reperfusion injury (CIRI) is a major contributor to poor prognosis of ischemic stroke. Flavonoids are a broad family of plant polyphenols which are abundant in traditional Chinese medicine (TCM) and have beneficial effects on several diseases including ischemic stroke. Accumulating studies have indicated that flavonoids derived from herbal TCM are effective in alleviating CIRI after ischemic stroke *in vitro* or *in vivo*, and exhibit favourable therapeutical potential. Herein, we systematically review the classification, metabolic absorption, neuroprotective efficacy, and mechanisms of TCM flavonoids against CIRI. The literature suggest that flavonoids exert potential medicinal functions including suppressing excitotoxicity, Ca^2+^ overloading, oxidative stress, inflammation, thrombin’s cellular toxicity, different types of programmed cell deaths, and protecting the blood-brain barrier, as well as promoting neurogenesis in the recovery stage following ischemic stroke. Furthermore, we identified certain matters that should be taken into account in future research, as well as proposed difficulties and opportunities in transforming TCM-derived flavonoids into medications or functional foods for the treatment or prevention of CIRI. Overall, in this review we aim to provide novel ideas for the identification of new prospective medication candidates for the therapeutic strategy against ischemic stroke.

## 1 Introduction

Stroke, which includes two subtypes of ischemia and haemorrhage, is the second leading cause of death globally (Wang et al., 2017). The occurrence of ischemic stroke is approximately four times that of hemorrhagic stroke in different geographical areas (Collaborators et al., 2018; Li Q. et al., 2019; Barthels and Das, 2020). Ischemic stroke occurs when there is a sudden obstruction in the flow of arterial blood to a specific area of the brain, resulting in localized neurological impairments. The prompt restoration of blood flow through revascularization is widely acknowledged as a highly effective method in alleviating cerebral ischemic injury. The primary treatment of choice for patients with ischemic stroke is thrombolysis or thrombectomy to re-canalize the occlusion and re-perfuse the cerebral, by either pharmacological or mechanical means (Qin et al., 2022). Currently, recombinant tissue-plasminogen activator is the sole medication sanctioned by the Food and Drug Administration for the treatment of ischemic stroke (Lahr et al., 2012; Mendelson and Prabhakaran, 2021). However, some of the limitations of intravenous thrombolysis are delays in achieving reperfusion, incomplete recanalization, hemorrhagic transformation, and secondary injury following reperfusion (Khandelwal et al., 2016). Of those, cerebral ischemia/reperfusion injury (CIRI) is a biochemical cascade that worsens brain tissue damage and offsets the benefits of restoring blood flow after an ischemic stroke. The pathogenesis of CIRI is complex, including oxidative stress, inflammation, apoptosis, blood-brain barrier (BBB) disruption, energy metabolism impairment, glutamate/neurotoxin release, calcium overload, and so on (Wu et al., 2017; Zhu et al., 2017; Al-Mufti et al., 2018; Wang J. F. et al., 2018; Wu et al., 2018). Therefore, the quest for safer and more effective natural remedies to prevent and treat CIRI is now a major research challenge.

Traditional Chinese medicines (TCM) have been used for thousands of years to treat various diseases in China. Recently, TCM has attracted more and more attention from international medical researchers due to its good therapeutic effect and low toxic side effects. Numerous studies indicated that rich varieties of flavonoids were widely contained in TCM, including flavans, flavones, flavonols, anthocyanidins, and so on. Flavonoids, natural polyphenol substances (Shen et al., 2022), have attracted attention for their strong anti-oxidant, anti-inflammatory, and neuroprotective activities (Ullah et al., 2020). A growing body of literature reports the use of botanical medicine flavonoids in the prevention and treatment of CIRI at the time of ischemic stroke. However, information on the potential mechanisms of action of botanical medicine flavonoids for CIRI prevention and treatment remains fragmented. Hence in this review, recent researchs on Chinese herbal medicine flavonoids against CIRI were systematically reviewed, including chemical structures, *in vivo* biotransformation, and pharmacological functions or mechanisms. This review aims to describe the research progress of how different Chinese herbal medicine flavonoids in treating CIRI to provide theoretical support for the application of those flavonoids in ischemic stroke.

## 2 Overview of Chinese herbal medicine flavonoids

### 2.1 Structural characterization

Flavonoids are polyphenol secondary metabolites with over 6,000 constituents (Shen et al., 2022). 30 flavonoids extracted from herb medicines have shown promise in treating CIRI. The basic skeleton of botanical medicine flavonoids consists of three rings (C6-C3-C6) in their chemical structure. According to its structure, Chinese herbal medicine flavonoids are classified into seven subclasses: flavonols, flavanols, flavones, isoflavones, anthocyanidins, flavanones, and chalcones (Shen et al., 2022). The core heterocycle’s oxidation degree determines this classification. Flavonols, also known as 3-hydroxyflavonoids, have several specific substitutions in rings A and B that are associated with a three-carbon chain. The positions of 5 and 7 on the Flavonol A ring were replaced by hydroxyl groups. Kaempferol, quercetin, galangin, icariside, and silymarin are anti-CIRI botanical medicine flavonols (Dabeek and Marra, 2019; Thapa et al., 2023; Luo et al., 2022; Xie et al., 2019). The flavone chemical structure consists of 4H-chromen-4-one with a phenyl substituent at position 2. Chinese herbal medicine flavones against CIRI include breviscapine, baicalein, ligustroflavone, chrysin, vitexin, hispidulin, scutellarin, acacetin, calycosin-7-O-β-D-glucoside, diosmetin, luteoloside, soybean isoflavones (SI), 3-daidzein sulfonate sodium, puerarine (Pur), ginkgetin, and astragalin (Patel et al., 2013; Zhou et al., 2014; Adnan et al., 2020; He et al., 2016; Yuan et al., 2016; Dinda et al., 2017; Yuan et al., 2017; Riaz et al., 2018; Naz et al., 2019; Zhang et al., 2019; Hu et al., 2020; Singh et al., 2020; Ashaq et al., 2021; Wen et al., 2021; Caporali et al., 2022; Xu et al., 2022). Isoflavones are a group of molecules that have a molecular structure based on the 3-phenyl chromen-4-one backbone. Genistein and formononetin are anti-CIRI isoflavones extracted from botanical medicine (Jaiswal et al., 2019; Machado et al., 2021). Anthocyanidins, glycosides of polyhydroxy and polymethoxy derivatives, tend to be unstable and commonly exist in the form of glycosylated anthocyanins. The arrangement of the hydroxyl and methoxyl groups as substituents in the flavylium structure generates different anthocyanins. Herbal anthocyanidins against CIRI only include procyanidins (PC) (Lee et al., 2022). Flavanones, dihydroflavones, have a saturated C ring. Flavanones are different from other subclasses because they are saturated with double bonds between C2 and C3. CIRI-fighting Chinese herbal medicine flavanones include astilbin, didymin, sikokianin A, and dihydromyricetin (Li et al., 2012; Yao et al., 2018; Zhang et al., 2018; Sharma et al., 2020). Flavanols, also known as flavanol-3-ol, have unique structural properties with a hydroxyl group attached to position 3 of the C-ring. Flavanols, in contrast to other subclasses, do not possess a double bond between positions 2 and 3. It is worth mentioning that the hydroxyl groups on the A, B, and C rings differ among various flavanols. The only flavanols derived from Chinese herbal medicine against CIRI are epigallocatechin-3-gallate (EGCG) (Cione et al., 2019). Chalcones are a class of compounds referred to as 1,3-diphenyl-2-propene-1-ones. They consist of two aromatic rings named as A and B rings which are linked by a three-carbon *α*, *β*-unsaturated carbonyl system. Besides, the A and B rings of chalcones contain three modified or unmodified C5-, C10-, and C15-prenyl moieties. Botanical medicine chalcones against CIRI only include Hydroxysaffor yellow A (HSYA) (Table 1) (Xue et al., 2021).

**TABLE 1:** Subclasses and chemical structures of Chinese herbal medicine flavonoids.

Subclasses	Compounds	Molecular formulas	Structures	Ref.
Anthocyanidin	Procyanidins	C_30_H_26_O_13_	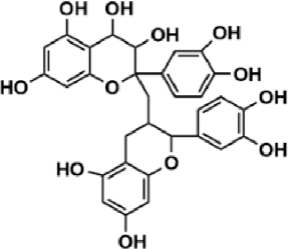	Lee et al. (2022)
Chalcone	Hydroxysaffor yellow A	C_27_H_32_O_16_	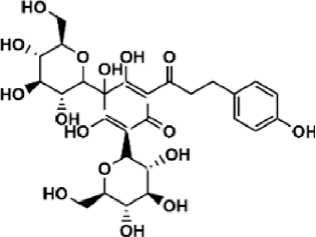	Xue et al. (2021)
Flavanol	Epigallocatechin-3-gallate	C_22_H_18_O_11_	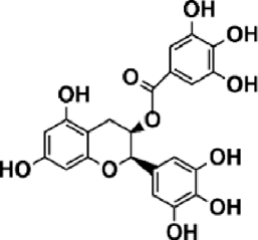	Cione et al. (2019)
	Astilbin	C_21_H_22_O_11_	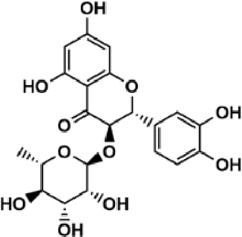	Sharma et al. (2020)
	Didymin	C_28_H_34_O_14_	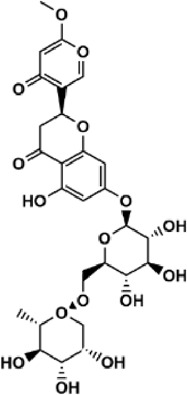	Yao et al. (2018)
	Dihydromyricetin	C_15_H_12_O_8_	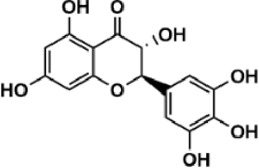	Zhang et al. (2018)
	Kaempferol 3-Rhamnoside	C_21_H_20_O_10_	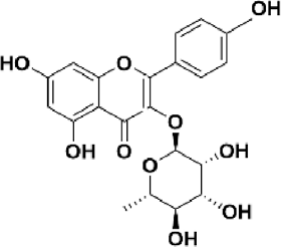	Lin et al. (2022)
	Sikokianin A	C_31_H_24_O_10_	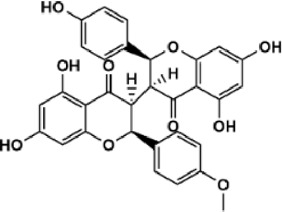	Li et al. (2012)
	3-daidzein sulfonate sodium	C_15_H_9_NaO_7_S	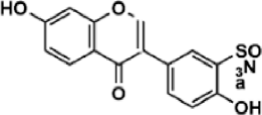	Yuan et al. (2017)
	Acacetin	C_16_H_12_O_5_	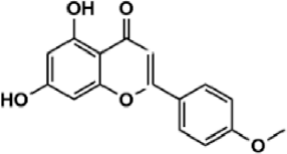	Singh et al. (2020)
	Astragalin	C_21_H_20_O_11_	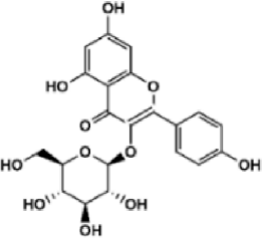	Riaz et al. (2018)
	Baicalein	C_15_H_10_O_5_	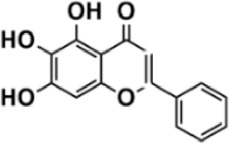	Dinda et al. (2017)
	Breviscapine	C_21_H_18_O_12_	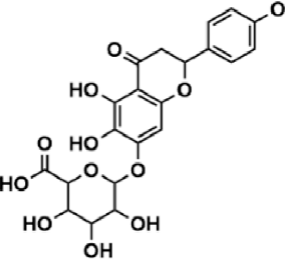	Wen et al. (2021)
	Calycosin-7-O-β-D-glucoside	C_22_H_22_O_10_	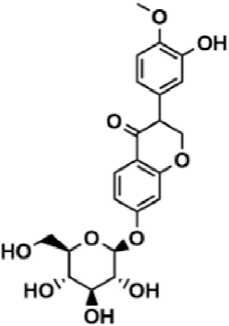	Xu et al. (2022)
	Chrysin	C_15_H_10_O_4_	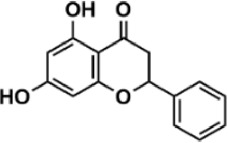	Naz et al. (2019)
	Diosmetin	C_16_H_12_O_6_	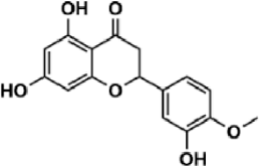	Patel et al. (2013)
	Ginkgetin	C_32_H_20_O_10_	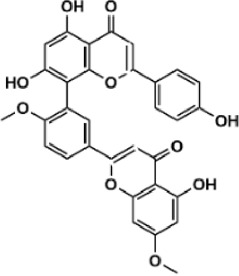	Adnan et al. (2020)
	Hispidulin	C_16_H_12_O_6_	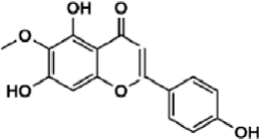	Ashaq et al. (2021)
	Ligustroflavone	C_30_H_40_O_18_	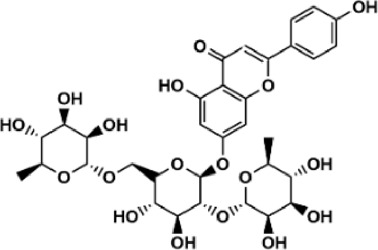	Zhang et al. (2019)
	Luteoloside	C_21_H_20_O_11_	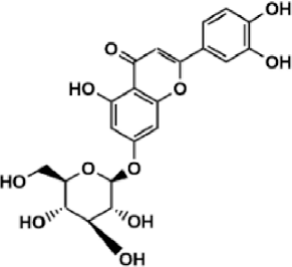	Caporali et al. (2022)
	Puerarine	C_21_H_20_O_9_	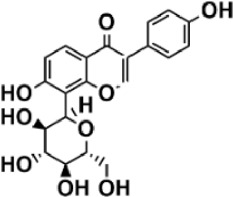	Zhou et al. (2014a)
	Scutellarin	C_21_H_18_O_12_	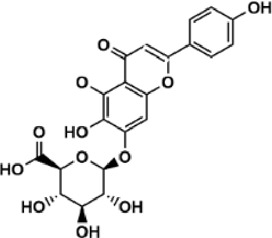	Yuan et al. (2016)
	Soybean isoflavones	C_46_H_32_O_14_	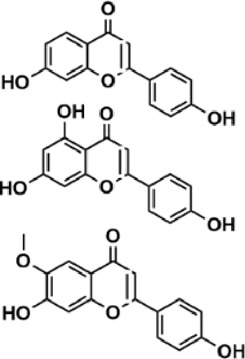	Hu et al. (2020)
	Vitexin	C_21_H_20_O_10_	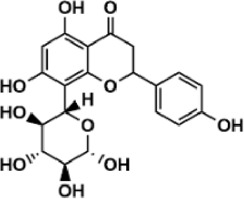	He et al. (2016)
Flavonol	Galangin	C_15_H_10_O_5_	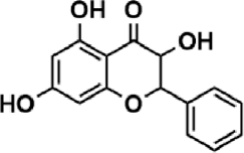	Thapa et al. (2023)
	Icariside	C_27_H_30_O_11_	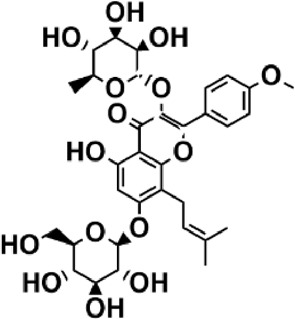	Luo et al. (2022)
	Kaempferol	C_15_H_10_O_6_	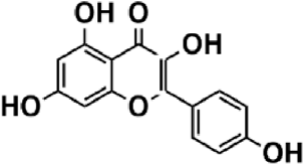	Dabeek and Marra. (2019)
	Myricetin	C_15_H_10_O_8_	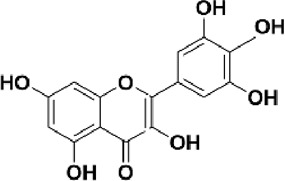	Wang et al. (2022)
	Quercetin	C_15_H_10_O_7_	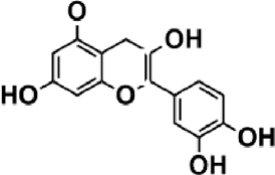	Dabeek and Marra. (2019)
	Silymarin	C_25_H_22_O_10_	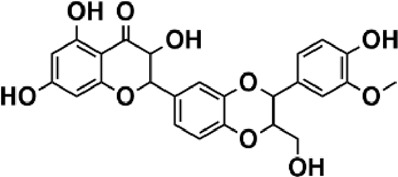	Xie et al. (2019)
Isoflavone	Formononetin	C_16_H_12_O_4_	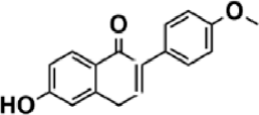	Machado et al. (2021)
	Genistein	C_15_H_10_O_5_	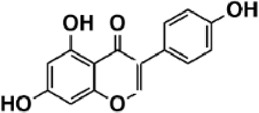	Jaiswal et al. (2019)
	Nobiletin	C_21_H_22_O_8_	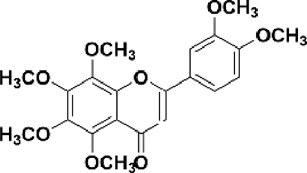	Yoshigai et al. (2013)

### 2.2 Metabolism *in vivo*

**FIGURE 1:**
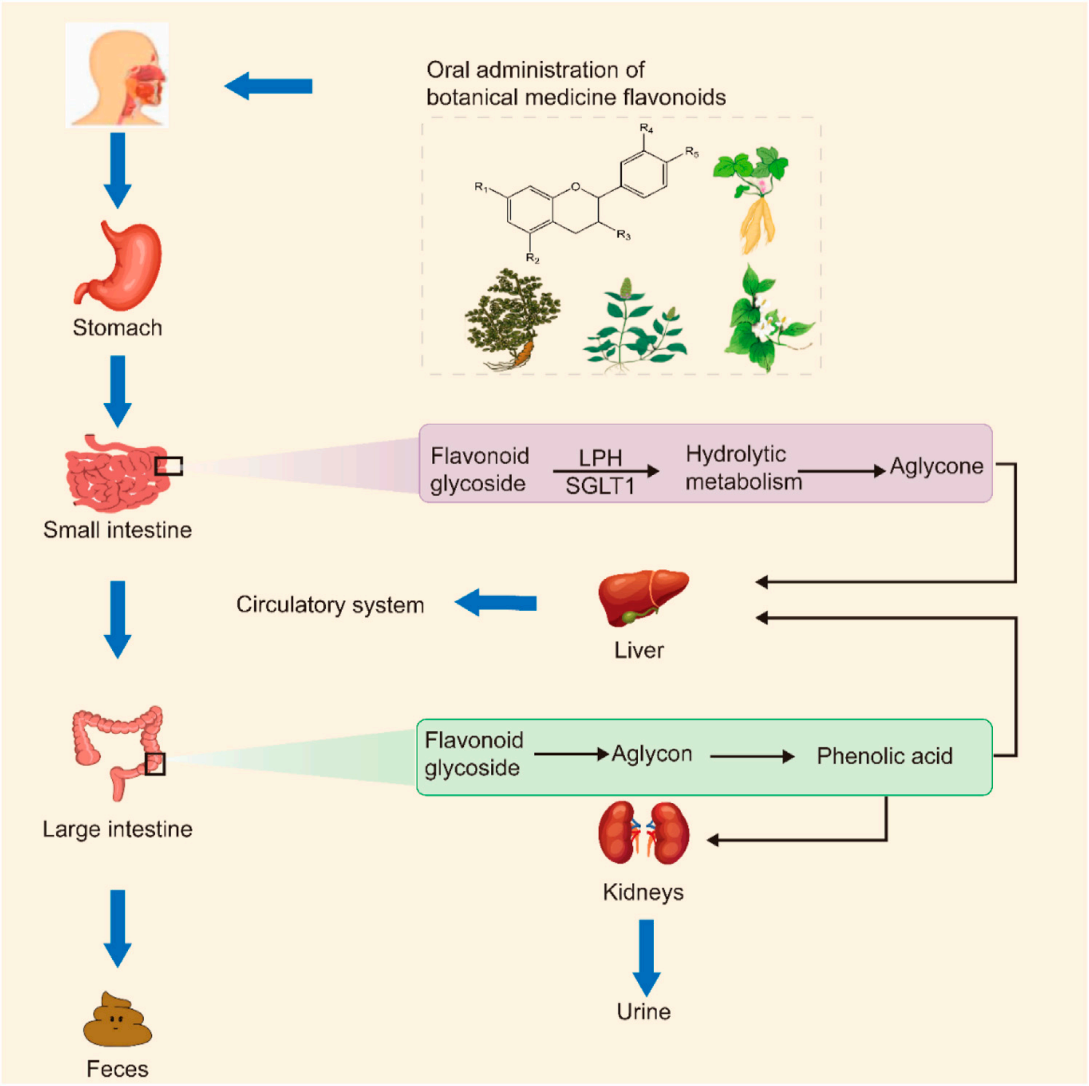
The fate of botanical medicine flavonoids *in vivo*. Upon gastrointestinal absorption, botanical medicine flavonoids undergo renal and fecal excretion, eliminating them from the body. Once absorbed by the small and large intestines, these bioactive compounds enter the systemic circulation, exerting their therapeutic potential on specific molecular targets within the human body, thus alleviating CIRI.

Flavonoids and their metabolites derived from botanical medicine possess antioxidant, anti-inflammatory, and immunomodulatory properties. Understanding the metabolic process of flavonoids is the key to further understanding their biological function. Once ingested orally, flavonoids derived from botanical medicine are taken up by the stomach, small intestine, liver, and colon. The biotransformation process occurs systemically. Flavonoids are metabolized and bioavailability is enhanced by enzymes found in the liver, small intestine, and large intestine, as well as gut microbes in the small intestine and colon. Due to the presence of phenolic hydroxyl groups, flavonoids derived from herb medicines are weakly acidic and can be absorbed in the stomach, which presents a weakly acidic environment (Crespy et al., 2002). Flavonoid glycosides are considered to be difficult to absorb in the small intestine due to greater hydrophilicity and relative molecular mass. In the small intestine, most flavonoid glycosides are absorbed into the body mainly through hydrolytic metabolism to glycosides in the presence of transport of sodium-glucose cotransporter 1 (SGLT1) or lactose-phlorizin hydrolase (LPH). Due to the relative hydrophobicity of flavonoid aglycone, this form of the compound can be more readily transported across membranes from the small intestine into the blood circulation via passive transportation (Tammela et al., 2004). After passing through selective absorption by both the stomach and small intestine, any botanical medicine flavonoids that remain unabsorbed, along with their metabolites, are excreted from the small intestinal wall and return to the intestinal cavity, where they pass through the peristalsis of the small intestine into the large intestine. *Enterobacter*’s enzymes (b-glucosidase, a-rhamnosidase, and b-glucuronidase) in the large intestine convert flavonoid glycosides into aglycons, which may then be converted into phenolic acid. The phenolic acid then enters into the systemic circulation and undergoes Phase Ⅱ metabolism in the liver and kidneys under the influence of Phase Ⅱ enzymes. Most of the flavonoids found in natural herbal plants go through oxidation and binding processes in the liver. The liver’s cytochrome P450 is responsible for the oxidation process. The binding process mainly refers to polar functional groups such as hydroxyl present in either the initial drug or the I-phase metabolite following oxidation. These groups are then coupled or combined with endogenous substances under enzyme catalysis, producing different binding products. In the liver, botanical medicine flavonoids bind through processes of glucuronization, sulfation, and methylation. Finally, the body eliminates Chinese herbal medicine flavonoids through excretion by the kidneys and in feces. Most of the excreted compounds are metabolites of flavonoids found in herbal medicines, while some are excreted unchanged (Figure 1). Still, shortcomings exist in pharmacokinetic investigations on herbal medicine flavonoids. The metabolic processes of the majority of monomeric flavonoids found in Chinese herbal medicine, along with their absorption and metabolic conversion mechanisms within the human body, remain incompletely understood. In addition, the role of flavonoids in phytomedicine and their interaction with other substances remains a focus of research. Further research is therefore needed.

## 3 Neuroprotective effects and mechanisms

**FIGURE 2:**
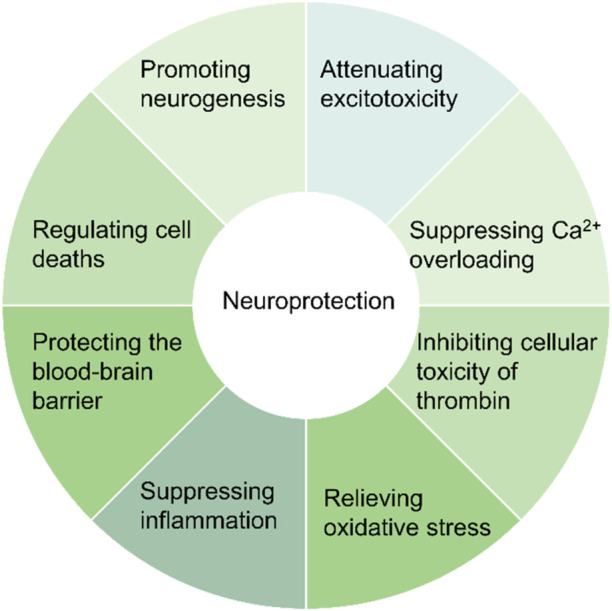
The neuroprotective efficacy of Chinese herbal medicine flavonoids. The botanical medicine flavonoids play a neuroprotective role by decreasing excitotoxicity, Ca^2+^ overloading, oxidative stress, inflammation, thrombin toxicity, and cell deaths, and protecting BBB and neurogenesis.

CIRI kills brain cells through inflammation, oxidative stress, ionic imbalance, and excitotoxicity (Jurcau and Simion, 2021). After ischemia, there is a disruption in ionic balance and excitotoxicity leading to cell death. Oxidative stress peaks during the initial reperfusion phase as a result of a drastic increase in the generation of reactive oxygen species (ROS) after oxygen is restored. Inflammation may endure for several days or weeks following reperfusion, resulting in delayed cell death following an ischemic stroke (Anrather and Iadecola, 2016). These processes may induce apoptosis, necrosis, ferroptosis, and pyroptosis in brain cells by directly or indirectly enhancing ER stress and dysfunction of mitochondria (Yang et al., 2018; Wang et al., 2022a; Wang et al., 2022b; Tuo et al., 2022). Herb medicine flavonoids can control the occurrence and development of CIRI by inhibiting oxidative stress, reducing inflammation, and inhibiting brain cell deaths, among other mechanisms (Figure 2). Herbal drug flavonoids may alleviate CIRI by targeting these biological pathways, preserving nerves, and improving neurological recovery.

### 3.1 Attenuating excitotoxicity

Excitotoxicity relates to the quick and significant release of the excitatory amino acid glutamate (Glu) and the inhibition of its reuptake due to energy deficits (Lai et al., 2014). Brain ischemia, hypoxia, and glucose deficiency cause disorders in neuronal energy metabolism, the release of glutamate, and depolarisation of the presynaptic membrane (Zhou et al., 2018). Furthermore, Glu stimulates excitatory amino acid receptors, predominantly the N-methyl-D-aspartate receptors (NMDARs). This process initiates the activation of calcium channels gated by receptors, culminating in an influx of calcium ions and subsequent elevation of intracellular calcium concentration (Yu et al., 2023). These events trigger a series of pathological responses in the cytoplasm and nucleus of the cell, ultimately leading to cytoskeleton destruction and neuronal necrosis (Lai et al., 2014). Furthermore, an excessive amount of calcium in mitochondria leads to their destruction, activating downstream apoptotic pathways ultimately resulting in apoptosis (Szydlowska and Tymianski, 2010). Therefore, attenuating excitotoxicity is essential for alleviating CIRI.

Many natural compounds, for instance, baicalin, HSYA, and Pur have been identified to exert a neuroprotective impact by blocking excitatory toxicity. Baicalin is a flavonoid extracted from *Scutellaria baicalensis Georgi*, which has been used for thousands of years in TCM. (Song et al., 2020) investigated the impact of baicalin on GS within cultured astrocytes. The observations demonstrated that baicalin enhanced glucose disposal by increasing GS activity, potentially due to upregulated GS expression instead of direct activation by baicalin. Furthermore, Liu and others researched the protective properties of baicalin in rat hippocampal slices against ischemic-like or excitotoxic injuries, as well as its impact on protein kinase C alpha (PKC-α) activation (Liu et al., 2005). Baicalin, at concentrations of 0.1, 1, and 10 mmol/L, provided effective protection against reductions in cell viability and acute swelling of neurons brought about by OGD, which was observed in a concentration-dependent manner. Additionally, it caused a reduction in the proportion of PKC-α present in the cell membrane fraction compared to the overall PKC-α count. At a concentration of 1 mmol/L, baicalin increased the decline in viability caused by NMDA (rather than the rise in LT) and lowered the membrane fraction of PKC-α, which was raised by NMDA. Thus, baicalin has been shown to prevent both ischemic and excitotoxic damage in slices of rat hippocampus. This positive outcome is attributed to the inhibition of translocation of PKC-α. *Carthamus tinctorius* L. produces a chemical compound known as HSYA, which studies indicate possesses neuroprotective effects in various pathological states, including cerebral ischemia. However, the precise way that HSYA impacts neurons is still unclear. To clarify the physiological mechanism responsible for the neuroprotective benefits of HSYA (Wang et al., 2016), conducted Ca^2+^ imaging, Western blotting, and mitochondrial targeted circularly permuted yellow fluorescent protein transfection experiments on cultured hippocampal neurons. By conducting electrophysiology experiments, it was determined that HSYA has the ability to hinder excitatory postsynaptic currents facilitated by NMDAR. This inhibition of NMDARs by HSYA was observed to be dependent on its concentration. HSYA also promotes excitatory postsynaptic currents mediated by paired NMDARs. Furthermore, HSYA reduced the membrane depolarisation currents induced by OGD that were mediated by NMDAR. In addition, it averted ischemic long-term potentiation, which is considered to be a factor in the progression of severe reperfusion injury after ischemia. In studies of molecular biology, it was discovered that HSYA inhibits the rise of intracellular calcium concentration triggered by NMDA and mediated by NMDAR in hippocampal cultures. Furthermore, it was observed to reduce the number of cells undergoing apoptosis and necrosis and alleviate mitochondrial damage. Thus, the data indicate that HSYA safeguards hippocampal neurons against excitotoxic damage through the inhibition of NMDARs. Pur (8-C-C-glucopyranosyl-4′-7-dihydroxy), an herb widely used in TCM, has been extracted from the dried root of *Pueraria lobata* (Wild) Ohwi (Xu et al., 2007). After an MCAO surgery, Pur was injected (50 and 100 mg/kg, i.p.) (Xu et al., 2007) observed the neuroprotective effects of Pur against neurotoxicity induced by Glu. The extracellular levels of Glu, Asp, GABA, and Tau induced by ischemia were significantly decreased in the striatum of animals treated with vehicle, with reductions of 54.7%, 56.7%, 75.8%, and 68.1%. Pur displayed a more prominent reduction in Glu and Asp levels compared to GABA and Tau, while the Glu/GABA ratio was significantly lower in rats treated with Pur during MCAO, compared to those treated with the vehicle. The Pur intervention resulted in a noteworthy decrease in apoptosis and necrosis instigated by Glu in hippocampal neurons cultured *in vitro*. Therefore, the acute administration of Pur at the onset of occlusion results in a neuroprotective effect on cellular viability. This effect is due to the significant reduction in ischemia-induced amino acid efflux, specifically excitotoxicity in the striatum. In short, flavonoids found in botanical medicine can reduce the excitotoxicity caused by CIRI by increasing GS and PKC-α levels and decreasing NMDARs, Asp, Glu, GABAT, and Tau.

### 3.2 Suppressing Ca^2+^ overloading

Excessive glutamate release after ischemic insults to neurons leads to elevated Ca^2+^ levels, resulting in the development of excitotoxicity (Yu et al., 2023). Ca (2+)/calmodulin-dependent protein kinase II (CaMKII is recognised as a key player in facilitating biochemical events that lead to cellular demise as a result of acute excitotoxic injuries (Coultrap et al., 2011). The neurons were protected from an excitotoxic injury by inhibiting CaMKII.

(Li et al., 2013) aimed to explore any potential correlation between the neuroprotective effects of baicalin on the ischemic area and CaMKII. The protein expression of CaMKII and p-CaMKII was evaluated in gerbils belonging to various treatment groups. The results indicate that there were no differences in total CaMKII levels among the various groups. Concurrently, the model group displayed augmented p-CaMKII expression in contrast to the sham-treated group; conversely, the model plus treatment group exhibited a decrease in expression. *In vitro,* the findings demonstrate that the OGD group showed a significant increase in p-CaMKII expression compared to the control group, whereas the OGD + baicalin group indicated a marked decrease (Wang et al., 2016). Nobiletin (NOB), a polymethoxylated flavonoid, is found in Citrus fruits. It is also known as 5,6,7,8,3′,4′-hexamethoxyflavone (Yoshigai et al., 2013). Administration of nobiletin at a dose of 50 mg/kg i.p. restored the reduced levels of CaMKII autophosphorylation and phosphorylation at Thr-34 of dopamine- and cAMP-regulated phosphoprotein-32 (DARPP-32) in the striatum and hippocampal CA1 to levels comparable to those observed in sham-operated mice (Yabuki et al., 2014). In addition, the nobiletin treatment prevented the reduction of CaMKII, MAP2, and GluR1 protein levels in the hippocampal CA1 region. This was accompanied by the restoration of both ERK and CREB phosphorylation and CaMKII autophosphorylation (Yamamoto et al., 2009). The study investigated the effect of Kaempferol 3-rhamnoside (KR), a flavonoid isolated from Schima superba with neuroprotective properties, on the release of glutamate from rat cerebrocortical nerve terminals. (Lin et al., 2022) demonstrated that KR inhibits glutamate release in rat cerebrocortical nerve terminals. This effect may be exerted primarily through the suppression of P/Q-type Ca^2+^ channels and CaMKII/synapsin I pathways. Tilianin is a flavonoid glycoside that occurs naturally and has various biological activities, such as antioxidant, anti-inflammatory, energy-collecting, and anti-hypoxic effects. The neuroprotective effects of tilianin against cerebral ischemia are achieved through the attenuation of CaMKII-linked signaling mediated through mitochondria and p38/JNK/NF-κB inflammatory pathways (Jiang et al., 2018). In summary, the results demonstrate that flavonoids found in botanical medicine can suppress Ca^2+^ overloading caused by CIRI by decreasing CaMKII.

### 3.3 Inhibiting cellular toxicity of thrombin

There exists a notable correlation between thrombin’s cellular toxicity and nerve damage attributed to cerebral ischemia due to thrombin’s ability to interact with the protease-activated receptor-1 (PAR-1) in cerebral tissues (Lyden et al., 2021). Protease-activated receptors are part of a superfamily of G-protein coupled receptors with seven transmembrane domains (Griffin et al., 2018). PAR-1 is frequently present in the hypothalamus, hippocampus, and cerebellum (Luo et al., 2007). Therefore, inhibiting PAR-1 is imperative in alleviating nerve damage resulting from cerebral ischemia.

To explore the neuroprotective properties of baicalin on Wistar rats subjected to focal CIRI, 90 rats were randomly divided into five groups. The baicalin groups exhibited a significant decrease in PAR-1 mRNA and PAR-1 expression when compared to the vehicle group. The high-dose group displayed a significant reduction in PAR-1 mRNA and PAR-1 level, in contrast to the low-dose baicalin group. Therefore, baicalin displayed neuroprotective effects in cases of CIRI by suppressing the expression of PAR-1 (Navarro-Núñez et al., 2009; Zhou et al., 2014) demonstrated that quercetin, apigenin, and genistein inhibit both PAR1-and PAR4-mediated platelet aggregation. However, they seem to be slightly less effective in inhibiting thrombin-induced aggregation. Astragalin (AG) is a flavonoid that occurs naturally and can be extracted from various edible plants, including green tea seeds, *Morus alba* L., and *Cuscuta chinensis*. AG has a wide range of pharmacological activities and can be used to treat various diseases, including cancers, osteoarthritis, osteoporosis, ulcerative colitis, mastitis, obesity, diabetes mellitus, diabetic complications, ischemia/reperfusion injury, neuropathy, respiratory diseases, and reproductive system diseases (Chen et al., 2023). The expression of PAR-1 and PAR-2 was induced by the model group, and this effect was attenuated by the administration of ≥10 mg/kg astragalin. According to (Kim et al., 2021), astragalin may disrupt the oxidative stress-MAPK signaling-inflammation axis by disconnecting PAR activation from thromboembolism. Myricetin is a flavonoid compound that occurs naturally in various fruits and vegetables. It has been found to possess excellent biological properties, including anti-inflammatory, antioxidative, and antitumor activities. (Wang et al., 2022) research show that myricetin inhibits PAR-1 activation. In summary, the results demonstrate that flavonoids found in botanical medicine can inhibit cellular toxicity of thrombin caused by CIRI by decreasing PAR-1.

### 3.4 Relieving oxidative stress

Oxidative stress, a critical determinant in the pathogenesis of CIRI (Li et al., 2018), is characterized by the abundance of ROS generation beyond cellular antioxidant power (Chen et al., 2020). When CIRI occurs, the quantity of ROS produced exceeds the rate at which they are eliminated. This may cause lipid oxidation, destruction of cellular and mitochondrial membranes, and potentially even neuronal death (Perluigi et al., 2012). Therefore, regulating the balance of oxidative stress by inhibiting ROS production and promoting their scavenging activity has proven to be an effective method of protecting neurons against CIRI (Wu et al., 2020). The inbuilt antioxidant system, which includes enzymes that metabolise free radicals like SOD, CAT, GSH, and GPx, effectively eliminates ROS in usual physiological circumstances. However, an increased need for antioxidants arises to mitigate oxidative stress, which occurs when ROS production exceeds the capacity of internal antioxidants during oxidative stress (Tang et al., 2017).

Numerous flavonoids, which are found in nature, function as exogenous antioxidants and enhance the effectiveness of antioxidant enzymes. Flavonoids like quercetin, sikokianin A, and Diosmetin, alleviate oxidative stress after CIRI. Quercetin, a significant flavonoid compound derived from *Taxillus sutchuenensis* (Lecomte) Danser (TS, Sangjisheng in Chinese), is among the quality control indexes of TS. It possesses a range of characteristics such as reducing blood pressure, anti-hyperlipidemia, anti-hyperglycemia, antioxidant, anti-inflammatory, neuroprotective, and cardio-protective properties (Hosseini et al., 2021). During their study (Guo et al., 2021), found that Quercetin has the potential to enhance the survival of neurons by reducing the production of NQO-1, HO-1, SOD1, and GPx1. Moreover, they observed that the Nrf2/HO-1 pathway is involved in the promotion of LDH levels. Likewise, baicalin stimulated the Nrf2/HO-1 pathway within neurons, improving the expression of its antioxidant genes HO-1 and NQO-1. This resulted in significant reductions in cerebral ischemic injury resulting from stroke (Huang et al., 2021). Sikokianin A, identified from *Wikstroemia indica* (L.) C.A. Mey (a medicinal plant), can reduce the elevated levels of ROS in PC cells. After OGD/R exposure, there was an increase in MDA levels, which then decreased as the dose of sikokianin A increased, resulting in lipid peroxidation products (Yao et al., 2019). Diosmetin is a bioflavonoid compound naturally abundant in *Dendranthema morifolium* (Ramat.) Tzvel (Ning et al., 2021). Previous research has indicated that diosmetin may be able to shield against oxidative stress in CIRI, yet the precise mechanisms behind this have yet to be fully elucidated. Consequently (Mei et al., 2022), examined how diosmetin influences CIRI and its mechanisms of action. The research has revealed that diosmetin decreased lactate dehydrogenase (LDH) and ROS levels, and concurrently increased the expression of T-Nrf2, N-Nrf2, SIRT1, NQO-1, and HO-1 proteins. Icariside II (ICS II) is a flavonol glycoside and the main component of *Epimedium rotundatum* K.S. Hao, a traditional Chinese medicinal plant employed for the treatment of osteoporosis, inflammation, and cancer (Xu et al., 2021). The study investigated the impact of ICS II on oxidative damage. Results demonstrated that ICS II mitigated the injury to PC12 cells caused by OGD/R, confirmed by increased cell viability and reduced LDH leakage. In addition, ICS II not only reduced the ROS levels, but also inhibited the excessive production of mitochondrial ROS and restored the mitochondrial membrane potential. Furthermore, it is important to note that the levels of Nrf2, NQO-1, and HO-1 were reduced due to OGD/R, but these changes were effectively reversed with the application of ICS II. Besides, ICS II prevented the decrease in SIRT3 and IDH2 levels induced by OGD/R. To sum up, their study showed that ICS II reduces oxidative harm by controlling the Nrf2/SIRT3 signalling pathway (Feng et al., 2018). To summarise, plant-based flavonoids decrease CIRI-caused harm from oxidative stress. It is achieved by activating NQO-1, LDH, Nrf2, HO-1, SIRT1, and SOD while reducing ROS, IDH2, SIRT3, MDA, and GPX1. These are involved in the Nrf-2/HO-1, SIRT1/Nrf2, and Nrf2/SIRT3 pathways.

### 3.5 Anti-inflammation

After cerebral ischemia, inflammation leads to secondary damage which promotes CIRI (Talma et al., 2016). Inflammation in the ischemic region can be attributed to several crucial cytokines, chemokines, NO, ROS, and MMPs (Lambertsen et al., 2012; Kawabori and Yenari, 2015; Jayaraj et al., 2019). Out of these, the most prevalent mediators of ischemic stroke are IL-1β, IL-6, and TNF-α, primarily produced by immune and endothelial cells (Jayaraj et al., 2019).

Flavonoids such as formononetin, carthamin yellow, vitexin, luteoloside, and Chrysin have been shown to alleviate inflammation following CIRI by reducing pro-inflammatory cytokines or improving anti-inflammatory ones. Formononetin is a phytoestrogen derived from the *C. tinctorius* L. (Tay et al., 2019). The research conducted by Yu et al. (2022) was to investigate how formononetin could potentially alleviate inflammation in CIRI. The findings indicate that formononetin significantly improved the neurological deficit and pathological condition of brain tissue in MCAO rat models. Moreover, formononetin lowered levels of cytokines IL-18, TNF-α, IL-6, and IL-1β. Additionally, formononetin reduced the protein levels of p-JAK2, p-STAT3, NLRP3, ASC, cl-Caspase-1, and cl-IL-1β. The researchers concluded that formononetin has anti-inflammatory properties as it inhibits the JAK2/STAT3 signalling pathway (Yu et al., 2022). Carthamin yellow (CY), which is also derived from *C. tinctorius* L., has been found to mitigate the effects of heart ischemia-reperfusion injury. Nevertheless, it remains uncertain whether CY can enhance ischemic stroke. (Guo et al., 2021) conducted a Western blotting analysis which indicated CY decrease levels of p-IκBα, p-NF-Κb, caspase-1, p65, IL-1β, and NLRP3 protein expressions in MCAO rats. Additionally, CY reduced TNF-α, IL-1β, and IL-6 in the serum. Therefore, the administration of CY inhibited the inflammatory response in rats subjected to MCAO. Vitexin (apigenin-8-C-glucoside) is an active ingredient in a wide range of TCM. It is a c-glycosylated flavone and found in various herb medicines such as bamboo and chaste tree or chaste berry (He et al., 2016; Jiang et al., 2018) investigated the role of vitexin in MCAO. The group subjected to MCAO and vitexin demonstrated decreased serum levels of IL-6 and THF-α, while IL-10 levels were elevated. Luteoloside, which is mainly extracted from *Loniceraj aponica* Thumb, has anti-inflammatory, antioxidant, anticancer, and cardioprotective properties, but its neuroprotective benefits have been little studied. The research carried out by Li et al. (2019) examines the defensive impact of luteoloside on cerebral ischemia and its mechanisms. The findings suggest that there is a noteworthy decline in neuroinflammation caused by I/R with luteoloside administration. This is backed by the decrease of IL-1β, TNF-α, iNOS, and COX-2 levels with MCAO. Furthermore, their findings indicate that luteoloside effectively inhibited the activation of NF-κB signalling, raised the level of PPARγ protein expression, and boosted Nrf2 nuclear accumulation in rats with MCAO. The results indicate that in rats afflicted with focal cerebral ischemia, luteoloside restrained NF-κB signalling and provided protection to the brain. Furthermore, PPARγ and Nrf2 significantly affected the anti-inflammatory properties of luteoloside. Chrysin is a bioactive compound found in *Oroxylum indicum* (L.) Vent (Naz et al., 2019). Chrysin markedly decreased the concentrations of TNF-α, IL-6, and IL-1β both *in vitro* and *in vivo* via the stimulation of the PI3K/Akt/mTOR signalling pathway (Li et al., 2019). In conclusion, Chinese herbal medicine flavonoids ameliorate inflammation induced by CIRI by suppressing pro-inflammatory cytokines such as IL-1β, IL-6, IL-10, IL-18, TNF-α, iNOS, and COX-2 through inflammation-related JAK2/STAT3, NF-κB-PPARγ-Nrf2, and PI3K/Akt/mTOR pathways.

### 3.6 Protection of blood-brain barrier

The blood-brain barrier (BBB) is semipermeable and dynamic, serving to separate the bloodstream from the central nervous system (CNS) (Yu et al., 2022; Peluffo et al., 2015). BBB comprises astrocytes, pericytes, adjacent neurons, and endothelial cells (Hawkins and Davis, 2005). The endothelial cells are connected by means of a junction complex, with a considerable contribution from the tight junction (TJ). By altering TJ, hypoxia, and ischemia leads to elevated permeability of the BBB (Sandoval and Witt, 2008). In cases of acute ischemic stroke, the blood-brain barrier becomes more permeable due to changes in the positioning of TJ proteins like occludin, claudin-5, and ZO-1 (Shin et al., 2016). In the I/R model, claudin-5, occludin, and ZO-1 levels decreased markedly, thereby leading to an elevation in BBB permeability (Sandoval and Witt, 2008). Several studies indicate that the increased activation of MMPs during ischemia leads to the degradation of extracellular matrix components and the TJ, which in turn affects the permeability of the BBB (Asahi et al., 2001). Therefore, it is crucial to increase the expression of TJs (occludin, claudin-5, and ZO-1) and decrease the expression of MMPs to repair BBB leakage in the I/R model. In addition, P-glycoprotein (P-gp), which is an ATP-driven drug efflux transporter, plays a critical role in the blood-brain barrier (Miller et al., 2008). After cerebral ischemia, P-gp is upregulated as an efflux transporter on microvascular endothelial cells. The degradation of endothelial tight junctions by elevated microvascular endothelial P-gp resulted in increased BBB hyperpermeability, accelerated brain inflammation, and ultimately worsened ischemic stroke outcomes (Huang et al., 2022). Therefore, it is also essential to inhibit the expression of P-gp to alleviate CIRI.

Several botanical flavonoids (baicalin, ICS II, and quercetin) possess the ability to restore the BBB function. The research discovered that LPS resulted in reduced amounts of TJ proteins within the BBB. Nevertheless, baicalin can alleviate such damage by augmenting the level of Claudin-5 and ZO-1 (Wang et al., 2021). The impact of ICS Ⅱ on the BBB was uncertain. Hence (Liu et al., 2020), investigated how ICS Ⅱ regulates BBB integrity in rats following CIRI and the underlying mechanisms. The findings indicate that ICS II was greatly efficient in averting BBB disruption, as revealed by the Evans Blue staining. Furthermore, the use of ICS II resulted in a substantial decrease in MMP2/9 expression while simultaneously increasing the expression of TIMP1 and TJ proteins (Liu et al., 2020). In rats subjected to MCAO, treatment with quercetin at doses of 10, 30, and 50 mg/kg demonstrated a capability to inhibit the decrease in TJ protein expression (ZO-1, occludin, and claudin-5), suggesting its prospective role in preserving BBB integrity by regulating the expression of TJ protein (Yang et al., 2022). At a concentration of 200 μM, baicalein (−)-epigallocatechin gallate, kaempferol, quercetin, and silymarin significantly increased the intracellular accumulation of rhodamine 123. This effect may be due to the inhibition of P-gp activity (Ferreira et al., 2018). Biochanin A, morin, phloretin, and silymarin inhibited P-gp-mediated cellular efflux. The mechanism of interaction involved a direct interaction, at least in part (Zhang and Morris, 2003). Procyanidine, a class of phenolic compounds, has been reported to exhibit a wide range of biological effects including antibacterial, antiviral, antiinflammatory, antiallergic and vasodilatory actions. The study shows that procyanidine was a potent inhibitor of P-gp on BBB and could improve the therapeutic effects on cerebral of some drugs which are difficult to accumulate in the brain (He et al., 2009). In summary, botanical flavonoids have the ability to mend disruptions to the BBB brought on by CIRI, as they elevate the expression of tight junctions (TJs)- specifically occludin, claudin-5 and ZO-1, and inhibit MMP2/9 and P-gp.

### 3.7 Regulating programmed cell deaths

#### 3.7.1 Autophagy

Autophagy is the degradation of proteins and organelles. In mammals, autophagy is necessary for cell maintenance, survival, differentiation, and development (Fleming et al., 2022). Basal autophagy operates as a neuronal “housekeeping” process, while induced autophagy represents a promising neuroprotective strategy for counteracting ischemic stroke (Mizushima and Komatsu, 2011). Various stress conditions, such as hypoxia and oxidative stress, typically generate signals that activate the autophagic process (Fleming et al., 2022). Autophagy involves the PI3K/Akt/mTOR and AMPK/mTOR pathways (Heras-Sandoval et al., 2014; Mihaylova and Shaw, 2011). The ULK1 complex is the central focus of these signalling pathways. It consists of ULK1, ATG13, FIP200, and ATG101 (Zachari and Ganley, 2017). The ULK1 complex induces phagophore nucleation through phosphorylation of components within PI3KC3 complex I (Wirth et al., 2013). VPS34, Beclin 1, ATG14, AMBRA1, and p115 facilitate the local generation of PI3P at a characteristic structure of the endoplasmic reticulum named the omegasome (Roberts and Ktistakis, 2013). Through the interaction of their PI3P-binding domains, PI3P recruits WIPI2 and DFCP1, which are PI3P effector proteins with WD repeat domains and double FYVE-containing domains respectively, to the omegasome (Yu et al., 2018). Recently, it was discovered that WIPI2 directly binds ATG16L1 and recruits the ATG12∼ATG5-ATG16L1 complex. This complex improves the ATG3-mediated conjugation of ATG8s, comprising microtubule-associated protein LC3 proteins and GABARAPs, with membrane-resident PE (Martens and Fracchiolla, 2020). As a result, membrane-bound, lipidated forms are created. In this conjugation reaction, LC3-I is converted into LC3-II, which serves as the unique marker for autophagic membranes. ATG8s play a pivotal role in extending and fusing the phagophore membrane by enlisting other constituents of the autophagic apparatus possessing an LC3-interacting region (LIR). In the context of selective autophagy, LC3 selectively transports cargo that is labelled into autophagosomes via cargo receptors containing LIR (Yu et al., 2018). The plasma membrane, mitochondria, recycling endosomes, and Golgi apparatus all supply membrane components necessary for the extension of the autophagosomal membrane. In addition, ATG9-containing vesicles provide certain lipid bilayers. The autophagosomal membrane, once sealed, gives rise to an autophagosome. As it matures, the ATG proteins are eliminated, and the autophagosome ultimately merges with the lysosome. Acid hydrolyses in the lysosome breakdown autophagic cargo, thus letting go of the nutrients to be utilized within the cytoplasm of the cell (Dikic and Elazar, 2018). Growing evidence indicates that autophagy is triggered in different cell types within the cerebral, such as neurons, glial cells, and cerebral microvascular cells, during an ischemic stroke (Wang et al., 2018). Therefore, the regulation of autophagy plays a crucial role in treating CIRI.

**FIGURE 3:**
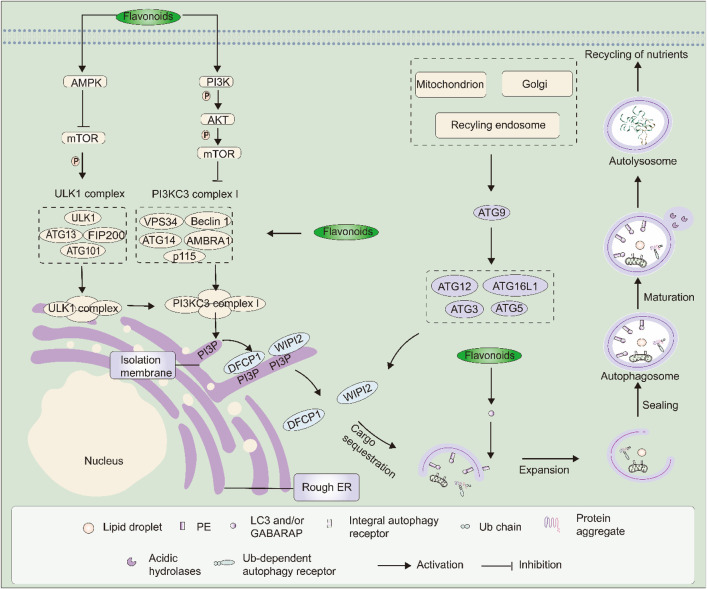
The signalling pathways and targets of botanical medicine flavonoids against CIRI-induced autophagy. Botanical medicine flavonoids alleviate CIRI-induced autophagy by upregulating LC3, LC3-Ⅱ, ULK1, AMPK, p-Akt, mTOR, Beclin1 and LC3-Ⅱ/LC3-Ⅰ ratio and downregulating LC3-Ⅱ/LC3-Ⅰ ratio, Beclin-1 through mediating the PI3K/Akt/mTOR pathway and AMPK-mTOR-ULK1 signalling pathway.

Several flavonoids from different botanical medicines were studied to determine their impact on autophagy induced by CIRI. Curcumin, which is derived from the *Curcuma longa* L., has attracted attention due to its powerful bioactive properties against damage caused by ischemia. Curcumin was administered intraperitoneally at a dose of 200 mg/kg, 30 min after I/R. 30 min prior to MCAO, a PI3K/Akt/mTOR pathway inhibitor called LY294002 was injected into the ventricle. Saline was administered as a control. Curcumin improved the level of Akt and mTOR and reduced LC3-Ⅱ/LC3-I expression in MCAO rats, resulting in enhanced brain injury recovery and neurological function (Huang et al., 2018). Pur is the primary active component obtained from *P. lobata* (Willd.) Ohwi. This plant is commonly known as Gegen in TCM, which has the effect of improving blood circulation. Its active ingredients can prevent cardiovascular and cerebrovascular diseases, and assist in the treatment of coronary heart disease, angina pectoris, headache, and other obvious effects. Pur (50 or 100 mg/kg) resulted in decreased levels of Beclin-1 expression and the LC3-Ⅱ/LC3-I ratio, along with reductions in p-AMPK and pS317-ULK1. In contrast, it increased the p62 expression. Pur (100 mg/kg) significantly elevated the expression of p-mTOR and pS757-ULK1 in the ischemic region. Their findings suggest that Pur alleviates autophagy through the AMPK-mTOR-ULK1 signalling pathway (Wang et al., 2018). Ginaton is an extract of *Ginkgo biloba* L (GB) (Rojas et al., 2012). The study uncovered that administering Ginaton at a dosage of 50 mg/kg resulted in a rise in Beclin1 and LC3-Ⅱ expression. Additionally, it upregulated AMPK, mTOR, and ULK1 and triggered autophagy in MCAO rats (Li et al., 2019). Silibinin (SLB), extracted from the seeds of *Silybinus laborinum L.*, is a widely used antioxidant. Neuronal cell death was induced by exposure to H_2_O_2_ (100 μM) for 30 min with or without SLB at the concentrations indicated. Whole-cell extracts were obtained, and Western blot analysis was performed on samples (each containing 30 μg protein) using antibodies against mTOR, LC3, Beclin-1, and β-actin as a loading control. SLB significantly reduced overactive autophagic processes triggered by H_2_O_2_ by downregulating autophagy-related proteins (Beclin-1, LC3-Ⅱ) in cortical neurons (Wang et al., 2016). The levels of mTOR and PPAR-γ expression significantly decreased, while Ulk1 expression increased after MCAO. The administration of Vitexin prevented the reduction in mTOR and PPAR-γ expression and enhanced Ulk1. In addition, vitexin was effective in reducing the expression of Beclin1 and p62 in MCAO model rats, while vitexin was found to alleviate the increased rate of LC3 Ⅱ/LC3 I after MCAO (Jiang et al., 2018). Breviscapine, a flavonoid that originates from the traditional Chinese herb *Erigeron abajoensis Cronquist*, has been frequently employed in the clinical treatment of cerebral stroke in China. In Pengyue et al.'s study, their research indicates a remarkable reduction in infarct volume, brain edema, and neurofunctional impairment following 7 days of breviscapine therapy. The ratio of LC3-II to LC3-I decreased significantly in the MCAO + breviscapine group (Pengyue et al., 2017). This finding indicates the successful inhibition of over-activated autophagy activity induced by CIRI through the use of breviscapine. Numerous edible and medicinal plants are found to contain kaempferol, a flavonol known for its anti-inflammatory, antioxidant, and antiapoptotic potential (Lagoa et al., 2009). The mechanism by which kaempferol affects CIRI requires further exploration. The study utilized an MCAO model on rats to establish injury caused by ischemia/reperfusion. The rats in the treatment group received oral doses of kaempferol (at 50, 100, and 200 mg/kg) 7 days prior to the surgery. After kaempferol treatment, there was a significant upregulation of the expression of autophagy-related genes, mainly ATG4, ATG5, and ATG7, as well as autophagy-related proteins (Beclin1 and p62) in the MCAO group. Similarly, as a component of TCM, kaempferol has been shown to enhance the p-AMPK/p-mTOR in MCAO rats. Additionally, dorsomorphin treatment, which acts as an AMPK inhibitor, was found to reverse kaempferol-induced autophagy (Yuan et al., 2022). In conclusion, the use of plant-derived flavonoids improves autophagy induced by CIRI. This is achieved by increasing the levels of LC3, LC3-Ⅱ, ULK1, AMPK, p-Akt, mTOR, Beclin1, and LC3-Ⅱ/LC3-I ratio while reducing the LC3-Ⅱ/LC3-I ratio and Beclin-1 through the facilitation of the PI3K/Akt/mTOR and AMPK-mTOR-ULK1 signalling pathways (Figure 3).

#### 3.7.2 PANoptosis and ferroptosis

Recently, studies have demonstrated that pyroptosis, apoptosis, and necroptosis (PANoptosis) have taken place in MCAO rats (Yan et al., 2022). MLKL is the ultimate executor of necroptosis and a vital intermediary between this process and pyroptosis (Zhan et al., 2021). The association between RIPK1-RIPK3 or the activation of cytosolic ZBP1 leads to the phosphorylation of MLKL, causing pores to form on the membrane and resulting in the induction of necroptosis. MLKL-mediated release of plasma and potassium ion efflux induces cellular stress and triggers NLRP3 activation (Malireddi et al., 2019). This subsequently leads to the formation of the inflammasome and cleavage of caspase-1, ultimately resulting in the canonical pathway of pyroptosis (Fu and Wu, 2023). ZBP1 is capable of directly activating the NLRP3 inflammasome (Zheng and Kanneganti, 2020). Moreover, the binding of TNF to TNFR on the cellular membrane leads to the assembly and activation of complex I, resulting in the formation of apoptosome complex (Bao and Shi, 2007). Caspase-8 of the ripoptosome complex facilitates the initiation of caspase-3 and -7, resulting in the execution of GSDME-mediated pyroptosis (Orning et al., 2019). Apoptosis and pyroptosis are connected through caspases and their downstream components. When death receptors trigger extrinsic apoptosis, FADD and caspase-8 are recruited to initiate the death-inducing signalling complex (DISC) (Yang, 2015). Afterward, activated caspase-8 is able to initiate caspase-3 and -7, ultimately resulting in the execution of GSDME-mediated pyroptosis. In the intrinsic apoptotic pathway, caspase-8 and caspase-1 are capable of cleaving the Bcl-2 family member Bid into the proapoptotic tBID, which in turn triggers mitochondrial outer membrane permeabilization (Broughton et al., 2009). This leads to the discharge of cytochrome c, resulting in the generation of apoptosome and triggering caspase-9, further enhancing the activation of caspase-3 and -7.

Inhibiting PANoptosis by Chinese herbal medicine flavonoids. Ligustroflavone, which is a component of *Ligustrum vulgare* L., has been identified (Kang et al., 2021). In MCAO rats, ischemic injury was evident as neurological deficit score and infarct volume were increased, and RIPK1, RIPK3, and MLKL/p-MLKL were found to be elevated. However, when administered 30 mg/kg of ligustroflavone by the intragastric route 15 min prior to the ischemic event, there was a noteworthy amelioration of neurological function observed in addition to a decrease in infarct volume and a reduction in the levels of proteins linked to necroptosis. Further research has shown that the connection between RIPK3, RIPK1, and MLKL was significantly enhanced, but the presence of ligustroflavone had a diminishing effect on its impact (Zhang et al., 2019). To study how baicalin can alleviate CIRI, researchers examined the AMPK signalling pathway, which controls NLRP3 inflammasome activity. *In vivo*, the activation of the NLRP3 inflammasome resulted in an increase in IL-1β, ASC, NLRP3, cleaved caspase-1, and IL-18 production. Baicalin treatment showed a dose-dependent reversal of these effects. Moreover, it has been discovered that the suppression of NLRP3 expression enhances the neuroprotective properties of baicalin. Furthermore, baicalin noticeably increased p-AMPK expression *in vitro*. When the AMPK pathway is obstructed, there is an increase in the expression of NLRP3 inflammasome. The research shows that baicalin at doses of 100 and 200 mg/kg decreases NLRP3 inflammasome and hinders pyroptosis by activating the AMPK signalling pathway both *in vivo* and *in vitro*, consequently, alleviating CIRI (Zheng et al., 2021). Hispidulin, a flavonoid found in *Plantago asiatica* L., possesses noteworthy properties for decreasing blood pressure (Gao et al., 2016). In a study conducted by An et al. (2019), the cell viability in OGD/R was slightly increased to 60% and 70% through the application of 5 μM and 10 μM hispidulin, respectively. Moreover, hispidulin significantly decreased caspase-1, NLRP3, ASC, IL-18, and IL-1β *in vitro*, which is comparable to the *in vivo*. Additionally, OGD/R induced inhibition of AMPK and activation of GSK3β, which were subsequently reversed by treatment with hispidulin (An et al., 2019). Currently, multiple studies are analyzing the regulation of apoptosis through flavonoids in botanical medicine. (Tao et al., 2017) measured the expression of caspase-3. It was demonstrated that Pur inhibits the accumulation of cleaved-caspase-3, but not caspase-3 in MCAO rats. In the research conducted by El Khashab et al. (2019), an increase in Bcl-2 levels and a decrease in both BAX and Hsp90 levels were observed upon exposure to chrysin (30 mg/kg), which coincided with elevated levels of Glu and Asp (El Khashab et al., 2019). Didymin is extracted from *Melissa officinalis* L. (Li et al., 2022). Didymin has been reported to provide protection against intracerebral hemorrhage. However, the mechanism through which didymin regulates CIRI remains largely uncertain. The study conducted by Li *et al.* indicates that didymin pretreatment effectively lowers apoptotic rates, reduces Bax and c-caspase-3, and increases Bcl-2 levels in PC12 cells subjected to OGD/R (Li et al., 2022). Moreover, the PPAR signalling pathway was stimulated by didymin, resulting in the upregulation of PPAR-γ and RXRA expression *in vitro*. Astilbin, a derivative of dihydro flavonol derived from *Smilax glabra* Roxb., exhibits antioxidant anti-inflammatory, and antiapoptotic properties (Chen et al., 2018; Li et al., 2020) investigated the influence of Astilbin on OGD-triggered cellular apoptosis. Following the administration of Astilbin, there was a noteworthy reduction in the number of cells undergoing apoptosis and the extent of apoptotic activity, coupled with a decrease in the rate of apoptosis. Furthermore, there was an increase in the expression of cleaved caspase-3 *in vitro*, yet Astilbin effectively inhibited the expression of cleaved caspase-3. The administration of astilbin led to heightened Bcl-2 levels, diminished Bax levels, and a decrease in the Bax/Bcl-2 ratio that was dependent on concentration. Furthermore, Astilbin suppressed the induced expression of FADD due to OGD treatment. Calycosin-7-O-β-D-glucoside (CG) is a representative component found in *Astragalus membranaceus* (Fisch.) Bunge. CG therapy effectively increases the expressions of SIRT1, FOXO1, PGC-1α, and Bcl-2 protein *in vitro*, while reducing the expression of Bax (Yan et al., 2019). Astragalin is also extracted from *A. membranaceus* (Fisch.) Bunge. To determine if the neuroprotective properties of astragalin are linked to the anti-apoptotic pathway, the expression of Bcl-Xl, Bax, and c-caspase-3 be detected. The study revealed a significant increase in Bax and c-caspase-3 expression alongside a significant decrease in Bcl-Xl expression in the I/R rats. Treatment with astragalin resulted in a significant reduction in the expression levels of both Bax and cleaved caspase-3 whilst concurrently increasing the levels of Bcl-Xl protein. The aforementioned findings suggest that astragalin has an antiapoptotic impact on I/R injury (Li et al., 2018). Scutellaria baicalensis stem-leaf total flavonoid (SSTF) extracted from *Scutellaria baicalensis* Georgi., it has been reported that SSTF has anti-inflammatory, antioxidant, and antiapoptotic effects (Shengkai et al., 2022). In the groups of SSTF (50, 100, and 200 mg/kg), a significant increase was observed in the number of cells that showed immunoreactivity to Bcl-2. Besides, the Bcl-2-immunoreactive cells increased significantly in the SSTF (50, 100, and 200 mg/kg) (Zhao et al., 2013). Diosmin (DM) is derived from *Citrus Aurantium* L. To investigate the neuroprotective effects of DM in MCAO rats, mice were divided into 5 groups, including the sham-operated group (0.9% NaCl), the tMCAO group (0.9% NaCl), the vehicle group (10% lactos), the low-dose group (50 mg/kg DM), and the high-dose group (100 mg/kg DM). Results indicate that there were significant differences between the high-dose group and Vehicle regarding Bcl-2. After administering DM (100 mg/kg), the expression of Bcl-2 mRNA showed significant upregulation systemically in MCAO rats. The level of Bcl-2 in the low-dose group was higher than in the vehicle. An increased expression of Bax in both the vehicle and tMCAO groups, which was significantly attenuated with treatment using a high dose of DM. Therefore, DM treatment markedly upregulated Bcl-2, and downregulated Bax *in vivo*, suggesting a strong antiapoptotic effect of DM in cerebral I/R (Liu et al., 2014). Icaritin (ICT) is a metabolite of icariin (ICA), both of which are extracted from *E. rotundatum* K.S. Hao. (horny goat weed), which enhances coronary blood flow, protects against myocardial ischemia, inhibits thrombosis, and promotes platelet production and aggregation, thereby improving cardiovascular function (Ma et al., 2011). Wu *et al.* analyze the expression of proteins related to apoptosis. In I/R mice, there was an increase in cleaved caspase-3, PARP, and Bax, accompanied by a substantial decrease in Bcl-2 expression. ICT treatment significantly reversed the cortical and hippocampal protein expression levels of these proteins (Wu et al., 2022). GB is a traditional Chinese medicinal herb which is used to treat cardiovascular diseases (Liu et al., 2018). Ginkgetin taken from GB leaves has shown promise in its ability to reduce inflammation and suppress the immune system. In a study conducted by Tian *et al.*, they demonstrated a connection between the protective effect of ginkgetin on the nerves and the prevention of apoptosis by stimulating the PI3K/Akt/mTOR signalling pathway (Tian et al., 2019). The findings suggest that the administration of ginkgetin led to a significant reduction in the volume of cerebral infarction and neurological deficits in I/R mice. Additionally, the treatment with ginkgetin reduced the quantity of apoptotic cells and decreased cleaved caspase-3 and Bax while simultaneously increasing the level of Bcl-2 in a dose-dependent manner. Moreover, the phosphorylations of Akt and mTOR were significantly enhanced by 100 mg/kg of ginkgetin. The antiapoptotic effect was weakened and the phosphorylation of Akt and mTOR was lowered due to the inhibition of PI3K by LY294002. Therefore, they have deduced that ginkgetin counteracts cerebral injury induced by ischemia-reperfusion by impeding apoptosis in rats. This outcome was weakened by the stimulation of the PI3K/Akt/mTOR signalling pathway. Researchers conducted a study where they induced a CIRI model in Kunming mice. The study measured extracellular signal-regulated kinases 1/2 (ERK1/2), c-Jun N-terminal kinases (JNK), and p38 phosphorylation and apoptosis. Western blot analysis showed that vitexin significantly increased p-ERK1/2 and decreased p-JNK and p-p38 expression levels. Furthermore, it was observed that vitexin augmented the expression of Bcl-2 while suppressing the overexpression of Bax in the I/R rat (Wang et al., 2015). The impact could potentially be regulated by the mitogen-activated protein kinase (MAPK) signalling pathways (Li et al., 2020). 3′-Daidzein sulfonate sodium (DSS) is a recently synthesised compound with augmented water solubility attained through the modification of the structure of daidzein. It is an active constituent of *P. lobata* (Willd.) Ohwi. Liu *et al.*'s study separated rats into five groups, and the immunohistochemistry results indicate that DSS (0.5, 1.0, and 2.0 mg/kg) significantly increased Bcl-2 expression in the cerebral area, while simultaneously inhibiting the expression of Bax and caspase-3. Treatment with DSS reduces the number of apoptotic cells. Additionally, the findings suggest that DSS administration at doses of 0.5, 1.0, and 2.0 mg/kg markedly increases Bcl-2 expression, while suppressing the level of caspase-3 and Bax and reducing the ratio of Bcl-2/Bax (Chen et al., 2013; Liu et al., 2017) analyze the effect of HSYA in apoptosis induced by CIRI and explore potential mechanisms. The rats underwent a 2-h occlusion period, followed by a 24-h period of reperfusion. The MCAO rats were injected HSYA via the tail vein 15 min following occlusion. The number of apoptotic cells, Bcl-2, Bax, Akt, and GSK3β in the ischemic penumbra were measured by The TUNEL assay and Western blot analysis. Based on the results, HSYA administered at 4 and 8 mg per kilogram resulted in a reduction in apoptotic cells and an increase in the ratio of Bcl-2/Bax in MCAO rats. Concurrently, treatment with HSYA significantly augmented the phosphorylation expressions of both Akt and GSK3β. However, Wortmannin effectively reduced the phosphorylation of Akt and GSK3β by blocking the expression of PI3K, thus nullifying its antiapoptotic impact. These findings indicate that HSYA provides partial protection against CIRI by decreasing apoptosis via activating PI3K/Akt/GSK3β signalling pathway. Hence, botanical medicine flavonoids alleviate CIRI-induced apoptosis by downregulating Hsp90, Akt1, GSK-3β, MCL-1, Bax/Bcl-2, Bax, FADD, caspase-3, and Bcl-2 through facilitating the SIRT1/FOXO1/PGC-1*α*, PI3K/Akt/mTOR, and PI3K/Akt/GSK3β signalling pathway.

A new form of cell death, termed ferroptosis, has been recently discovered. This phenomenon is characterised by the incidence of iron and lipid peroxidation, with the former being pivotal to the onset of ferroptosis. Ferroptosis-inducing substances have the ability to reduce antioxidant capacity and increase ROS in cells by affecting GPx through various pathways (Li et al., 2020). Recent research has revealed a strong correlation between ferroptosis and the pathophysiological mechanisms of CIRI (Li et al., 2021).

**FIGURE 4:**
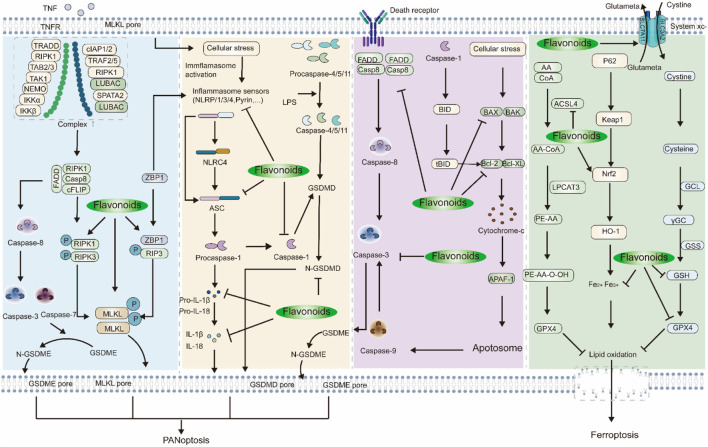
The signalling pathways and targets of botanical medicine flavonoids against CIRI-induced PANoptosis and ferroptosis. The inhibitory effect of botanical medicine flavonoids on PANoptosis and ferroptosis can potentially alleviate CIRI. Botanical medicine flavonoids play a neuroprotective role through increasing necroptosis-associated proteins (RIPK1, RIPK3, and MLKL/p-MLKL), thus alleviating CIRI. Chinese herbal medicine flavonoids repair CIRI-induced pyroptosis by down-regulating the expressions of NLRP3, ASC, cleaved caspase-1, IL-1β, IL-18, and GSDMD N-terminus. Botanical medicine flavonoids alleviate CIRI-induced apoptosis by downregulating Bax/Bcl-2, Bax, FADD, caspase-3, and Bcl-2. Moreover, Chinese herbal medicine flavonoids alleviate CIRI-induced ferroptosis by upregulating the SLC7A11 and GPX4.

Research has demonstrated that a variety of flavonoids can reduce the risk of CIRI by suppressing ferroptosis. Li *et al.* conducted research investigating the potential role of baicalein in CIRI using OGD/R cells, HT22 cells, and tMCAO mice (Li et al., 2022). Baicalein was found to be effective in decreasing iron levels, lipid peroxidation generation, and brain tissue ferroptosis morphology in MCAO, suggesting that it could reduce CIRI by restraining ferroptosis both in OGD/R HT22 cells and tMCAO mice. Additionally, they verified that baicalein exhibited the ability to suppress ferroptosis in HT22 cells. The Western blot analysis demonstrated that baicalein suppressed ferroptosis by controlling the levels of GPX4, ACSL4, and ACSL3 *in vivo* and *in vitro*. The study shows that baicalein has the ability to combat CIRI through anti-ferroptosis, which is regulated by the GPX4/ACSL4/ACSL3 axis (Xie et al., 2022). *Sophora japonica* L. is a rich source of kaempferol (Yuan et al., 2021) investigated the impact of kaempferol on ferroptosis *in vitro*. In neurons, the study demonstrated that OGD/R decreases in the levels of SLC7A11, GPX4, NADPH, GSH, and SOD. OGD/R significantly increased lipid peroxidation, ultimately leading to the initiation of ferroptosis in neurons. After kaempferol treatment, Nrf2/SLC7A11/GPX4 signalling was activated, leading to an increase in antioxidant capacity and a suppression of lipid peroxidation accumulation *in vitro*. In addition, kaempferol was able to significantly reduce the ferroptosis caused by OGD/R. However, inhibiting Nrf2 using ML385 hindered kaempferol’s protective abilities on antioxidant capacity, lipid peroxidation, and ferroptosis *in vitro*. Thus, kaempferol activates the Nrf2/SLC7A11/GPX4 signalling pathway to protect against ferroptosis *in vitro*. Galangin, a type of polyphenolic compound, is a flavonoid derived mainly from Chinese medicinal herbs, such as *Plantago major* L., *Languas officinarum* (Hance) Farw., and *Scutellaria galericulata* L. (Zhang et al., 2013). In the investigation carried out by Guan *et al.* Male gerbils, with a weight range of 70–90 g (12 weeks), were carefully chosen (Guan et al., 2021). The gerbils were provided with nourishment in a communal space, adhering to a 12-h cycle of alternating light and dark, while maintaining a controlled humidity and temperature of 22°C. Food and water were abundant. Fifty gerbils were randomly allocated into five groups. The sham group underwent an identical surgical procedure, but without carotid artery ligation, and was administered an equal amount of physiological saline to that of the treated groups. The groups of model + galangin underwent an identical procedure as the model group, and subsequently, received galangin (25, 50, and 100 mg/kg) for a continuous duration of 2 weeks. The administration of galangin was found to reduce lipid peroxide levels in brain tissue. Additionally, galangin treatment increased the level of SLC7A11 and GPX4. In addition, galangin-treated gerbils showed inhibition of levels of a marker of ferroptosis, while the effect of galangin was reduced when SLC7A11 was knocked down. By increasing the production of SLC7A11 and GPX4, the results show that galangin can prevent ferroptosis (Guan et al., 2021). PC is rich in nature and present in different plant species. To investigate whether PCs provide protection against CIRI, researchers utilised Western blotting to identify the presence of ferroptosis-related proteins including GPX4, SLC7A11, and TFR1. GPX4 and SLC7A11 are the primary proteins that combat lipid peroxidation, whereas TFR1 is closely associated with the Fenton reaction. GPX4 and SLC7A11 were expressed at a lower level in the I/R group compared to the sham group. In contrast, TFR1 was expressed at a higher level. The PC treatment group exhibited an elevation in the expression of GPX4 and SLC7A11, whilst the level of TFR1 was reduced. They ascertained the amount of Fe^2+^, MDA, and GSH as well. The Fe^2+^ and MDA concentrations rose in the I/R group yet dropped when PC was administered. The I/R group experienced a decline in GSH levels, whereas PC administration led to an increase. The data implies that administering PC had a positive effect on ferroptosis in CIRI mice. The expression of Nuclear-Nrf2 was significantly increased by CIRI, whereas HO-1 was downregulated. However, PCs had a substantial impact on the upregulation of Nuclear-Nrf2 and HO-1 expression. Their research demonstrates a means of mitigating CIRI-induced ferroptosis and protecting against damage to neuron cells by stimulating the Nrf2/HO-1 pathway (Chen et al., 2023). Daidzein is a phytoestrogen SI found in soybeans and other legumes (Alshehri et al., 2021). 120 male SD rats were assigned equally to the Sham group, CIRI group, and SI group. The levels of Fe^2+^, GSH, MDA, and MPO were detected by spectrophotometric tests in the ischemic penumbra. The expression of Fe^2+^ level, MDA level, GSH, and GPX4 in I/R rats was all significantly lower than that of Sham rats. However, all of these alterations were considerably improved in rats that received IS. Consequently, SI preconditioning diminishes CIRI in rats, potentially hindering ferroptosis (Li et al., 2023). In a word, Chinese herbal medicine flavonoids alleviate CIRI-induced ferroptosis by upregulating the SLC7A11 and GPX4 expressions and downregulating the expression of Fe^2+^, MDA, GSH, ROS, ACSL4, GPX4, and TFR1 through mediating the GPX4/ACSL4/ACSL3 axis, SPHK1/mTOR signalling pathway, Nrf2/SLC7A11/GPX4 signalling pathway, and Nrf2/HO-1 signalling pathway (Figure 4).

### 3.8 Promoting neurogenesis

Neurogenesis pertains to the generation of fresh neurons through neural stem/progenitor cells involving their proliferation, migration, and transformation into mature neurons (Schölzke and Schwaninger, 2007). Neurogenesis is generally agreed to occur mainly in the subventricular zone (SVZ) of the middle and lateral ventricles of the adult brain, as well as in the sub-granular zone (SGZ) of the hippocampal dentate gyrus (Wang and Jin, 2015). Under normal physiological conditions, there is a limited level of neurogenesis in the SVZ and SGZ areas. Nevertheless, in pathological conditions such as ischemic stroke, neural stem cells (NSCs) significantly proliferate in the SVZ region, migrate to the ischemic penumbra, and differentiate into mature neurons and glial cells, promoting neurological recovery (Libert et al., 2008). Newly generated neurons are significant in cognitive processes such as learning and retaining information, neuroplasticity, and emotional regulation. Neurogenesis provides a lot of promise for numerous stroke survivors. Therefore, ongoing research on cerebral ischemic injury focuses on neurogenesis and the improvement of neurological function.

Extensive research was conducted on the impact of different botanical medicine flavonoids on neurogenesis. Epigallocatechin-3-gallate is the primary component of green tea. The long-term impacts of EGCG on neurogenesis and functional recovery post-ischemic stroke remain uncertain. In the research conducted by Zhang *et al.*, MCAO was administered to C57BL/6 mice for a period of 60 min, followed by reperfusion for 28 days. The potential impacts of EGCG on both the proliferation and differentiation of neural progenitor cells (NPCs) were conducted through both *in vivo* and *in vitro*. *In vitro*, NPCs from the SVZ region were stimulated with LPS to enhance the inflammatory response subsequent to an ischemic stroke. It was discovered that the administration of EGCG for a duration of 14 days led to a notable enhancement in the growth of NPCs from the SVZ region, the movement of SVZ neuroblasts, and the restoration of functionality, potentially attributed to the induction of the M2 phenotype in microglia. After examining multiple signaling pathways, it was discovered that the AKT signaling pathway is involved in EGCG-induced proliferation and neuronal differentiation of NPCs *in vitro*. Their findings reveal a previously uncharacterised function of EGCG in facilitating the proliferation of SVZ and the differentiation of neurons (Zhang et al., 2017). Astragaloside IV is a crucial component present in extracts of *A. membranaceus* var. *Purpurinus* Y.C. Ho. Its vital biological properties include acting as an antioxidant, an anti-inflammatory, and an antiviral agent. In the research conducted by Chen *et al.*, Astragaloside VI was hypothesized to target the EGF-mediated MAPK signalling pathway to promote adult neurogenesis and regeneration after stroke. They examined the impact of Astragaloside VI on the stimulation of NSCs proliferation and self-renewal *in vitro*, as well as the augmentation of neurogenesis to facilitate the restoration of neurological functions in post-ischemic brains *in vivo*. *In vivo*, the rats were administered 1.5 h of MCAO alongside a 7-day reperfusion period. Astragaloside VI was administered intravenously at a dosage of 2 μg/kg per day for a duration of 7 consecutive days. *In vivo,* the administration of Astragaloside VI resulted in the promotion of neurogenesis and astrogenic formation in the SVZ, dentate gyrus zone, and cortex of the tMCAO. Without affecting differentiation, treatment with Astragaloside VI promoted the self-renewal and proliferation of NSCs *in vitro*. The Western blot analysis showed an augmentation in the size of neurospheres along with elevated levels of nestin, p-EGFR, and p-MAPK in response to Astragaloside VI. However, these effects were abrogated when combined with gefitinib (an EGF receptor inhibitor) and PD98059 (an ERK inhibitor). Behavioral experiments have revealed that the administration of Astragaloside VI resulted in a remarkable enhancement of both spatial learning and memory, as well as a significant improvement of motor function that had been previously impaired in tMCAO rats. Consequently, Astragaloside VI has the potential to effectively trigger the EGFR/MAPK signalling pathway, thereby improving the proliferation and neurogenesis of neural stem cells in tMCAO rats and enhancing the restoration of neurological functions in MCAO rats. This promising treatment avenue warrants further exploration (Chen et al., 2019). To explore the possible impact of baicalin on early neuron generation and stimulation of adult neurogenesis, the study found that treatment with baicalin significantly increased the number of cells that were double-positive for BrdU and NeuN in the subgranular zone (SGZ) of the hippocampal dentate gyrus (Zhuang et al., 2013). This observation bolsters their *in vitro* study that proposes baicalin’s ability to encourage the differentiation of proliferating NSPCs into a neuronal destiny. The outcomes suggest that baicalin has the potential to prompt the formation of fresh neurons post-cerebral ischemic damage (Zhuang et al., 2013). To summarize, flavonoids in botanical medicine enhance neurogenesis through the stimulation of NPC proliferation and the expression of VEGF, BDNF, and Mash1 via the AKT, MAPK, and EGFR/MAPK signalling pathways.

## 4 Conclusion and perspective

TCM’s efficacy and low toxicity have attracted attention. Chinese herbal medicine flavonoids have received considerable attention for their antiviral, anti-inflammatory, and antioxidant properties. Flavonoids found in oriental herb medicine undergo metabolism within the gastrointestinal tract and are subsequently absorbed into the body. They are then distributed through the bloodstream to multiple organs to perform their therapeutic roles. The botanical medicine flavonoids can prevent and treat CIRI by decreasing excitotoxicity, Ca^2+^ overloading, oxidative stress, inflammation, thrombin toxicity, and different types of programmed cell deaths, and protecting BBB and neurogenesis (Table 2). It appears that Chinese herbal medicine flavonoids may be a viable option for treating or preventing CIRI, either as a therapeutic agent or a dietary supplement. However, there remain several issues and debates regarding the investigation into the part and mechanism of Chinese herbal medicine flavonoids in the prevention and treatment of CIRI. Consequently, further research is necessary.

**TABLE 2:** Therapeutic effects of TCM-derived flavonoids against CIRI.

Compounds	Models	Regulation of targets	Effects or functions	Ref.
**3′-daidzein sulfonate sodium**	CIRI rats	↑Bcl-2 ↓Bax, ↓ Caspase-3	Inhibit apoptosis	Liu et al. (2017)
**Astilbin**	MCAO rats	↑Caspase-3, ↓Bax/Bcl-2, ↓FADD	Repress apoptosis	Li et al. (2020b)
**Astragalin**	BALB/c mice	↓PAR-1, ↓PAR-2	Inhibit cellular toxicity of thrombin	Kim et al. (2021)
**Astragalin**	I/R rats	↓Bax, ↓caspase-3, ↑Bcl-Xl	Inhibit apoptosis	Chen et al. (2020)
**Astragaloside VI**	Stroke rat	↑NSCs	Activate EGFR/MAPK, promote neurogenesis	Chen et al. (2019)
**Baicalein, (−)-epigallocatechin gallate, kaempferol, quercetin and silymarin**	MDCK-MDR1 cells	↓P-gp	Protect the blood-brain barrier	Ferreira et al. (2018)
**Baicalein**	OGD/R and tMCAO	↓GPX4, ↓ACSL4, ↓ACSL3	Mediate GPX4/ACSL4/ACSL3 signalling pathway, inhibit apoptosis	Li et al. (2022b)
**Baicalin**	OGD/R and MACO/R rats	↑GS	Combate excitotoxicity	Song et al. (2020)
**Baicalin**	OGD/R and MACO/R rats	↓NMDARs, ↑PKC-α	Attenuate excitotoxicity	Liu et al. (2005)
**Baicalin**	OGD/R and ischemic hippocampus	↓CaMK Ⅱ	Suppressing Ca^2+^ overloading	Li et al. (2013)
**Baicalin**	CIRI rats	↓mRNA, ↓PAR-1, ↓Caspase-3	Inhibit cellular toxicity of thrombin	Zhou et al. (2014b)
**Baicalin**	MACO rats	↑Claudin-5, ↑ZO-1, ↓MMP-9	Protect the blood-brain barrier	Wang et al. (2021)
**Baicalin**	MCAO rats	↓NLRP3, ↓ASC, ↓Caspase-1, ↓IL-1β, ↓IL-18	Activate AMPK signalling pathway, repair pyroptosis	Zheng et al. (2021)
**Baicalin**	tMCAO rats	↓HO-1, ↓NQO-1, ↑SOD1, ↑GPx1	Activate the Nrf2/HO-1 signalling pathway, relieve oxidative stress	Huang et al. (2021)
**Baicalin**	tMCAO rats	↑Mash1	Promote neurogenesis	Zhuang et al. (2013)
**Biochanin A, morin, phloretin, and silymarin**	MCF-7 and MDA435/LCC6	↓P-gp	Protect the blood-brain barrier	Zhang and Morris. (2003)
**Breviscapine**	MCAO rats	↓LC3Ⅱ/LC3Ⅰ	Regulate autophagy	Pengyue et al. (2017)
**Calycosin-7-O-β-D-glucoside**	OGD/R	↑Bcl-2, ↓Bax	Activate SIRT1/FOXO1/PGC-1α signalling pathway, inhibit apoptosis	Yan et al. (2019)
**Carthamin yellow**	CIRI rats	↓Fe^2+^, ↓ROS, ↓ACSL4, ↓GPX4	Inhibiting ferroptosis	Guo et al. (2021a)
**Carthamin yellow**	MACO rats	↓TNF-α, ↓IL-1β, ↓IL-6	Activate NF-κB/NLR signalling pathway, suppressing inflammation	Guo et al. (2021b)
**Chrysin**	CIRI rats	↑Bcl2, ↓BAX, ↓Hsp90	Repress apoptosis	El Khashab et al. (2019)
**Chrysin**	I/R rats	↓IL-6, ↓IL-1β, ↓TNF-α	Activate PI3K/Akt/mTOR signalling pathway, suppress inflammation	Li et al. (2019a)
**Curcumin**	I/R rats	↑p-Akt, ↑p-Mtor, ↓LC3-Ⅱ/LC3-Ⅰ, ↓IL-1, ↓TLR4, ↓p-38, ↓p-p38	Mediate the PI3K/Akt/mTOR pathway, regulate autophagy	Huang et al. (2018)
**Didymin**	OGD/R and CIRI rats	↓Bax, ↓c-caspase-3, ↑Bcl-2	Inhibit apoptosis	Li et al. (2022a)
**Dihydromyricetin**	CIRI rats	↓SPHK1, ↓mTOR	Inhibit SPHK1/mTOR signalling pathway, inhibiting ferroptosis	Xie et al. (2022)
**Diosmetin**	OGD/R and MCAO rats	↓NQO-1, ↓HO-1	Activate the SIRT1/Nrf2 signalling pathway, relieve oxidative stress	Mei et al. (2022)
**Diosmin**	tMCAO rats	↑Bcl-2, ↓Bax	Suppress apoptosis	Liu et al. (2014)
**Epigallocatechin-3-gallate**	Stroke rat	↑SZV	Regulate AKT signalling pathway, promote neurogenesis	Zhang et al. (2017)
**Formononetin**	MACO rats	↓IL-18, ↓TNF-α, ↓IL-6, ↓ IL-1β	Inhibit the JAK2/STAT3 signalling pathway, suppressing inflammation	Yu et al. (2022)
**Galangin**	CIRI rats	↑SLC7A11, ↑GPX4	Inhibiting ferroptosis	Guan et al. (2021)
**Ginaton**	MCAO rats	↑Beclin1, ↑LC3-Ⅱ, ↑AMPK, ↑mTOR, ↑ULK1	Inducing autophagy	Rojas et al. (2012)
**Ginkgetin**	CIRI rats	↓Caspase-3, ↓Bax, ↑Bcl-2	Repress apoptosis	Tian et al. (2019)
**Hispidulin**	OGD/R and MCAO rats	↓NLRP3, ↓ASC, ↓caspase-1, ↓IL-1β, ↓IL-18	Regulate AMPK/GSK3β signalling pathway, repair pyroptosis	An et al. (2019)
**Hydroxysafflor yellow**	CIRI rats	↑Bcl-2, ↓Bax	Regulate PI3K/Akt/GSK3β signalling pathway, inhibit apoptosis	Chen et al. (2013)
**Hydroxysafflor yellow A**	OGD/R and MACO/R rats	↓NMDARs	Inhibit excitotoxicity	Wang et al. (2016a)
**Icariside Ⅱ**	MCAO/R rats	↓Caspase-3	Inhibit the caspase 3-dependent apoptosis pathway, protect the blood-brain barrier	Liu et al. (2020)
**Icariside Ⅱ**	OGD/R	↑Nrf2, ↑SIRT3, ↓NQO-1, ↓HO-1	Activate the Nrf2/SIRT3 signalling pathway, relieve oxidative stress	Feng et al. (2018)
**Icaritin**	I/R mices	↑Caspase3, ↑PARP, ↑Bax, ↓Bcl-2	Suppress apoptosis	Wu et al. (2022)
**Kaempferol**	MCAO rats	↑LC3, ↑ Beclin-1, ↑p62, ↑AMPK, ↑mTOR	Regulate autophagy	Yuan et al. (2022)
**Kaempferol**	OGD/R	↑Nrf2, ↑SLC7A11, ↓GPX4	Regulate Nrf2/SLC7A11/GPX4 signalling pathway, inhibiting ferroptosis	Yuan et al. (2021)
**Kaempferol 3-rhamnoside**	Rat cerebrocortical nerve terminals	↓Q-type Ca^2+^ channels, ↓MKII/synapsin I pathways	Suppressing Ca^2+^ overloading	Lin et al. (2022)
**Ligustroflavone**	MCAO rats	↑RIPK1, ↑RIPK3, ↑ MLKL/p-MLKL	Repair necroptosis	Zhang et al. (2019)
**Luteoloside**	MACO rats	↓IL-1β, ↓TNF-α, ↓INOS, ↓COX-2, ↓PPARγ, ↑Nrf2	Suppress NF-κB signalling, suppress inflammation	Li et al. (2019b)
**Nobiletin**	Bilateral common carotid arteries occlusion mice	↓CaMKII, ↓MAP2, ↓GluR1	Suppressing Ca^2+^ overloading	Yamamoto et al. (2009)
**Nobiletin**	Parkinson model mice	↓CaMK Ⅱ	Suppressing Ca^2+^ overloading	Yabuki et al. (2014)
**Procyanidine**	Rat brain microvessel endothelial cells and nude mice transplanted with human cerebroma	↓P-gp	Protect the blood-brain barrier	He et al. (2009)
**Procyanidins**	CIRI rats	↑GPX4, ↑SLC7A11, ↓TFR1	Activate Nrf2/HO-1 signalling pathway, inhibiting ferroptosis	Cheng et al. (2023)
**Puerarin**	I/R rats	↑Caspase-3	Inhibit apoptosis	Tao et al. (2017)
**Puerarin**	MCAO rats	↓Beclin-1, ↓LC3-Ⅱ/LC3-Ⅰ, ↑p-mTOR, ↑pS757-ULK1	Activate AMPK-mTOR-ULK1 signalling pathway, regulate autophagy	Wang et al. (2018b)
**Puerarine**	MACO rats	↓Asp, ↓Glu, ↓GABA, ↓Tau	Alleviate excitotoxicity	Xu et al. (2007)
**Quercetin**	MACO/R rats	↓ROS, ↓NQO-1, ↓HO-1, ↓SOD1, ↓GPx1, ↑LDH	Activate the Nrf2/HO-1 signalling pathway, relieve oxidative stress	Guo et al. (2021a)
**Quercetin**	MCAO rats	↑Nrf2, ↑HO-1	Reduce cerebral infarct volume, neurological deficit, BBB permeability, and ROS generation via Sirt1/Nrf2/HO-1 signalling pathway	Yang et al. (2022)
**Quercetin, apigenin and genistein**	Venous blood from healthy volunteers	↓PAR-1, ↓PAR-4	Inhibit cellular toxicity of thrombin	Navarro-Núñez et al. (2009)
**Scutellaria baicalensis**	MACO rats	↑Bcl-2, ↓Bax	Suppress apoptosis	Zhao et al. (2013)
**Sikokianin A**	OGD/R	↑Nrf2, ↓HO-1	Activate the Nrf2/HO-1 signalling pathway, relieve oxidative stress	Yao et al. (2019)
**Silymarin**	MCAO rats	↑Beclin-1, ↑LC3-Ⅱ/LC3-Ⅰ, ↓H_2_O_2_	Activate PI3K/Akt-1/mTOR/Beclin-1 signalling pathway, regulate autophagy	Wang et al. (2016a)
**Soybean isoflavones**	MCAO rats	↓Fe^2+^, ↑MDA, ↓GSH ↓GPX4	Inhibiting ferroptosis	Li et al. (2023)
**Tilianin**	OGD/R	↓CaMK Ⅱ	Suppressing Ca^2+^ overloading	Jiang et al. (2018)
**Vitexin**	I/R injury mice	↑Bcl-2, ↓Bax	Repress apoptosis	Wang et al. (2015)
**Vitexin**	MACO rats	↓IL-6, ↓TNF-α, ↑IL-10	Suppress inflammation	Jiang et al. (2018a)
**Vitexin**	MCAO rats	↑Ulk1, ↑Beclin1, ↑LC3Ⅱ/LC3Ⅰ	Regulate mTOR/Ulk1 signalling pathway, suppress autophagy	Jiang et al. (2018b)

Large clinical trials of botanical medicine flavonoids. Many studies have examined the role of Chinese herbal medicine flavonoids in relieving CIRI, but their analysis of the underlying mechanisms is superficial at best. Thus, research is needed to find drugs that are selective, effective, and have few side effects. Large-scale clinical studies are needed into the clinical application of botanical flavonoids, including optimal dose and long-term safety.

The specificity and selectivity of flavonoids in botanical medicine for treating diseases. As is well known that Chinese herbal medicine flavonoids possess numerous potential targets, yet their intricate composition and absence of specificity and selectivity hinder their widespread utilization. Recently, bioinformatics combined with computer-aided drug discovery and design techniques have become an integral part of the development of small therapeutic compounds. We believe bioinformatics can be used to identify the exact pharmacological targets for the neuroprotection of flavonoids in natural herbs and to design and develop more effective neuroprotective agents.

The bioavailability of botanical medicine flavonoids. The primary flavonoids found in oriental herb medicine exhibit limited solubility in water, thereby diminishing their bioavailability. The bioavailability of flavonoids in Chinese herbal medicine can be improved through molecular modification, the combination of various active components, and orientation.

## Figures and Tables

**Table:** 

**Asp**	aspartate
**ATG8s**	ATG8 family proteins
**ATG13**	autophagy-related protein 13
**AMBRA1**	activating molecule in Beclin 1-regulated autophagy protein 1
**BBB**	blood-brain barrier
**CIRI**	cerebral ischemia/reperfusion injury
**CAT**	catalase
**CaMKⅡ**	Ca^2+^/calmodulin-dependent protein kinase Ⅱ
**CG**	calycosin-7-O-β-D-glucoside
**CNS**	central nervous system
**COX-2**	cyclooxygenase-2
**CY**	carthamin yellow
**Dex**	dexamethasone
**DFCP1**	double FYVE-containing protein 1
**DISC**	death-inducing signalling complex
**DM**	diosmin
**DSS**	3′-daidzein sulfonate sodium
**EGCG**	epigallocatechin-3-gallate
**EGF**	epidermal growth factor
**ER**	endoplasmic reticulum
**ERK**	the extracellular signal-regulated kinase
**GABA**	γ-amino butyric acid
**GABARAPs**	γ-aminobutyric acid receptor-associated proteins
**GB**	ginkgo biloba L
**Glu**	glutamate
**GPX**	glutathione peroxidase
**GPX4**	glutathione peroxidase 4
**GSH**	glutathione
**HSYA**	hydroxysafflor yellow A
**LC3**	light chain 3
**LDH**	lactate dehydrogenase
**LIR**	LC3-interacting region
**LPH**	lactose-phlorizin hydrolase
**LPS**	lipopolysaccharide
**JNK**	c-Jun N-terminal kinases
**ICA**	icariin
**ICS Ⅱ**	Icariside Ⅱ
**ICT**	icaritin
**IL-1β**	interleukin-1β
**IL-6**	interleukin-6
**I/R**	ischemia-reperfusion
**iNOS**	inducible nitric oxide synthase
**OGD**	oxygen/glucose deprivation
**OGD/R**	oxygen-glucose deprivation/reperfusion
**MAPK**	mitogen-activated protein kinase
**MCAO**	middle cerebral artery occlusion
**MMPs**	matrix metalloproteinases
**MOMP**	membrane permeabilization
**NPCs**	neural progenitor cells
**NADPH**	nicotinamide adenine dinucleotide phosphate
**NF-κB**	Nuclear factor-kappa B
**NMDA**	N-Methyl-D-Aspartate
**NMDAR**	N-methyl-D-aspartate receptors
**Nrf-2**	NF-E2-related factor
**NSCs**	neural stem cells
**PANoptosis**	pyroptosis, apoptosis and necroptosis
**PAR-1**	protease-activated receptor-1
**PC**	procyanidins
**PE**	phosphatidylethanolamine
**PI3P**	phosphatidylinositol-3-phosphate
**PI3KC3**	the class ⅡI PI3K
**PKC-α**	protein kinase C alpha
**PPARγ**	peroxisome proliferator-activated receptor gamma
**Pur**	puerarin
**r-tPA**	recombinant tissue plasminogen activator
**ROS**	reactive oxygen species
**SGLT1**	sodium-glucose cotransporter 1
**SI**	soybean isoflavone
**SLB**	silibinin
**SOD**	superoxide dismutase
**SGZ**	sub-granular zone
**SSTF**	scutellaria baicalensis stem-leaf total flavonoid
**SVZ**	subventricular zone
**Tau**	taurine
**TNF-α**	tumor necrosis factor-α
**TJ**	tight junction
**ULK1**	Unc-51-like kinase 1
**VPS34**	vacuolar protein sorting 34; ZO-1, Zonula occludens-1
